# Breaking Mental Barriers Promotes Recovery After Spinal Cord Injury

**DOI:** 10.3389/fnmol.2022.868563

**Published:** 2022-07-07

**Authors:** Haven I. Rodocker, Arman Bordbar, Molly J. E. Larson, Rebecca G. Biltz, Lynde Wangler, Paolo Fadda, Jonathan P. Godbout, Andrea Tedeschi

**Affiliations:** ^1^Department of Neuroscience, Wexner Medical Center, The Ohio State University, Columbus, OH, United States; ^2^Institute for Behavioral Medicine Research, The Ohio State University, Columbus, OH, United States; ^3^Department of Cancer Biology, The Ohio State University, Columbus, OH, United States; ^4^Chronic Brain Injury Program, The Ohio State University, Columbus, OH, United States

**Keywords:** spinal cord injury, gabapentinoids, mental health, neurogenesis, group intervention, functional recovery

## Abstract

Functional recovery after spinal cord injury (SCI) often proves difficult as physical and mental barriers bar survivors from enacting their designated rehabilitation programs. We recently demonstrated that adult mice administered gabapentinoids, clinically approved drugs prescribed to mitigate chronic neuropathic pain, recovered upper extremity function following cervical SCI. Given that rehabilitative training enhances neuronal plasticity and promotes motor recovery, we hypothesized that the combination of an aerobic-based rehabilitation regimen like treadmill training with gabapentin (GBP) administration will maximize recovery in SCI mice by strengthening synaptic connections along the sensorimotor axis. Whereas mice administered GBP recovered forelimb functions over the course of weeks and months following SCI, no additive forelimb recovery as the result of voluntary treadmill training was noted in these mice. To our surprise, we also failed to find an additive effect in mice administered vehicle. As motivation is crucial in rehabilitation interventions, we scored active engagement toward the rehabilitation protocol and found that mice administered GBP were consistently participating in the rehabilitation program. In contrast, mice administered vehicle exhibited a steep decline in participation, especially at chronic time points. Whereas neuroinflammatory gene expression profiles were comparable between experimental conditions, we discovered that mice administered GBP had increased hippocampal neurogenesis and exhibited less anxiety-like behavior after SCI. We also found that an external, social motivator effectively rescues participation in mice administered vehicle and promotes forelimb recovery after chronic SCI. Thus, not only does a clinically relevant treatment strategy preclude the deterioration of mental health after chronic SCI, but group intervention strategies may prove to be physically and emotionally beneficial for SCI individuals.

## Introduction

Severe impairments to physical and mental facets required for independent living are among the most devastating neurological deficits experienced following spinal cord injury (SCI) in adulthood. In about half of individuals living with SCI, the injury is sustained at the cervical level (Ahuja et al., 2017), leaving survivors to deal with long-term physical disability and profound psychological distress (Post and van Leeuwen, 2012). Currently, there are no readily translatable therapeutic strategies aimed at restoring neurological function available for SCI individuals.

Spinal cord repair strategies center upon the promotion of neuronal plasticity, axon regeneration and the formation of *de novo* neural circuits (Bradbury and McMahon, 2006; Tedeschi and Bradke, 2017; Hutson and Di Giovanni, 2019). By strengthening synaptic connections across the neuroaxis, research in the field also aims to boost compensatory mechanisms with the goal of restoring function (Nishimura et al., 2007). Among different rehabilitation strategies, cardiovascular-based protocols have been utilized to enhance neuronal plasticity, promote motor recovery and counteract cardiovascular diseases and associated complications (Krassioukov et al., 2019; Jo and Perez, 2020).

We recently discovered that administration of gabapentinoids (i.e., pregabalin and gabapentin), clinically approved drugs prescribed to treat neurological disorders such as chronic neuropathic pain (Rosner et al., 1996; Fornasari, 2017), promoted structural plasticity and regeneration of sensory ascending and descending motor pathways after SCI and ischemic stroke in adult mice (Tedeschi et al., 2016, 2022; Sun et al., 2020). However, the extent to which gabapentinoids administration may synergize with cardiovascular-based efforts like treadmill training is unknown. It is also unclear whether gabapentinoids may beneficially impact hallmarks of SCI psychopathology associated with deterioration of mental health, including anxiety and major depressive disorders.

In this study, we show that mice administered gabapentin (GBP) recovered forelimb functions over the course of weeks and months after cervical SCI, but no additive effect of voluntary treadmill training was noted in these mice in the context of increased forelimb recovery. To our surprise, we also failed to find an additive effect in mice administered vehicle. By scoring active engagement toward the rehabilitation protocol, we found that mice administered GBP were consistently engaged in the treadmill training regimen. In contrast, mice administered vehicle exhibited a decline in participation, especially at chronic time points when deterioration of mental health is more pronounced. Neuroinflammatory gene expression profiles within the lesion site did not show major differences between the two experimental conditions. Instead, we discovered that SCI mice administered GBP had enhanced hippocampal neurogenesis and were less subjected to anxiety-like behavior. We also found that an external, social motivator effectively rescues participation in control mice and promotes forelimb recovery after chronic SCI. Thus, not only does a clinically relevant treatment strategy prevent deterioration of mental health after chronic SCI, but group intervention strategies may beneficially integrate into the design of translational research to maximize neurological recovery in SCI survivors.

## Materials and Methods

### Mice

Adult (7- to 8-week-old) female and male C57BL/6J mice (stock no. 000664, The Jackson Laboratory) were used for all experiments. Mice were housed in groups of 4–5 per cage on a 12/12-h light-dark cycle with access to food and water ad libitum. Mice were randomly assigned to experimental groups. Experimenters were blind to group assignment and experimental conditions.

### Antibodies and Immunohistochemistry

The following antibodies were used: mouse monoclonal anti-NeuN (MAB377, RRID:AB_2298772, Millipore), rabbit polyclonal anti-GFAP (Z0334, RRID:AB_10013382, Dako), goat polyclonal anti-TrkB (AF1494, RRID:AB_2155264, R&D System), guinea pig polyclonal anti-doublecortin (AB2253, Millipore), goat polyclonal anti-Sox 2 (AF2018, R&D System) and mouse monoclonal anti-BrdU (MCA2483, RRID:AB_808349, Biorad). The mice were perfused and the cervical spinal cords and brains dissected and sequentially dehydrated in 10, 20, and 30% sucrose. Tissues were then embedded in optimum cutting temperature (OCT) compound (Tissue-Tek), frozen, sectioned (30–40 μm thick, HM525 NX, Thermo Fisher Scientific) and mounted on slides. Slides were warmed at 37°C for 30 min and OCT was washed away with PBS. Sections were then blocked at room temperature with 2.5% bovine serum albumin (A3059, Sigma-Aldrich) in PBS with 0.1% Triton-X100 for 1 h and incubated overnight at 4°C with the primary antibody. For BrdU immunohistochemistry, HCl pretreatment (2N HCl for 30 min at room temperature) was applied after the blocking step. After washing 3 times with PBS, sections were incubated with Alexa Fluor–conjugated secondary antibodies (1:500, Life Technologies). Sections were counterstained with DAPI (1:10,000, D9542, Sigma-Aldrich). Images were taken using a confocal (C2 plus, Nikon) and an inverted microscope (Axio Observer Z1, Zeiss) and analyzed using ImageJ (NIH) (Schindelin et al., 2012). Independent biological replicates (3 or more sections/mouse, *n* = 4–5 biological replicates) were analyzed.

### Cervical Spinal Cord Injury

Adult mice were anesthetized with a mixture of ketamine (100 mg/kg body weight) and xylazine (10 mg/kg body weight). A C5 laminectomy was performed and the spinal cord was crushed with No. 5 modified forceps (11254-20, Dumont, FST) as described previously (Sun et al., 2020). The forceps were positioned to completely sever half of the spinal cord. After injury, muscle layers were sutured and the skin was stapled using wound clips. To prevent dehydration, 0.9% saline solution was administered subcutaneously immediately after surgery. Postoperative care consisted of analgesic (meloxicam) administration (subcutaneous, every 24 h) during the first 3 postoperative days. All surgeries were conducted by the same experienced (blinded) investigator. Beginning 1 h after injury, GBP (46 mg/kg body weight, PHR1049, CAS:60142-96-3, MilliporeSigma) or the corresponding volume of vehicle (0.9% sodium chloride, 0409-7138-09, Aqualite system) was administered (intraperitoneal injections, 3 times/day for the first week, 2 times/day until the end of the study).

### Behavioral Testing

With the exception of the elevated zero maze, all mice were habituated to the behavioral setups prior to collection of baseline measures.

#### Treadmill Training

**Figure 1:**
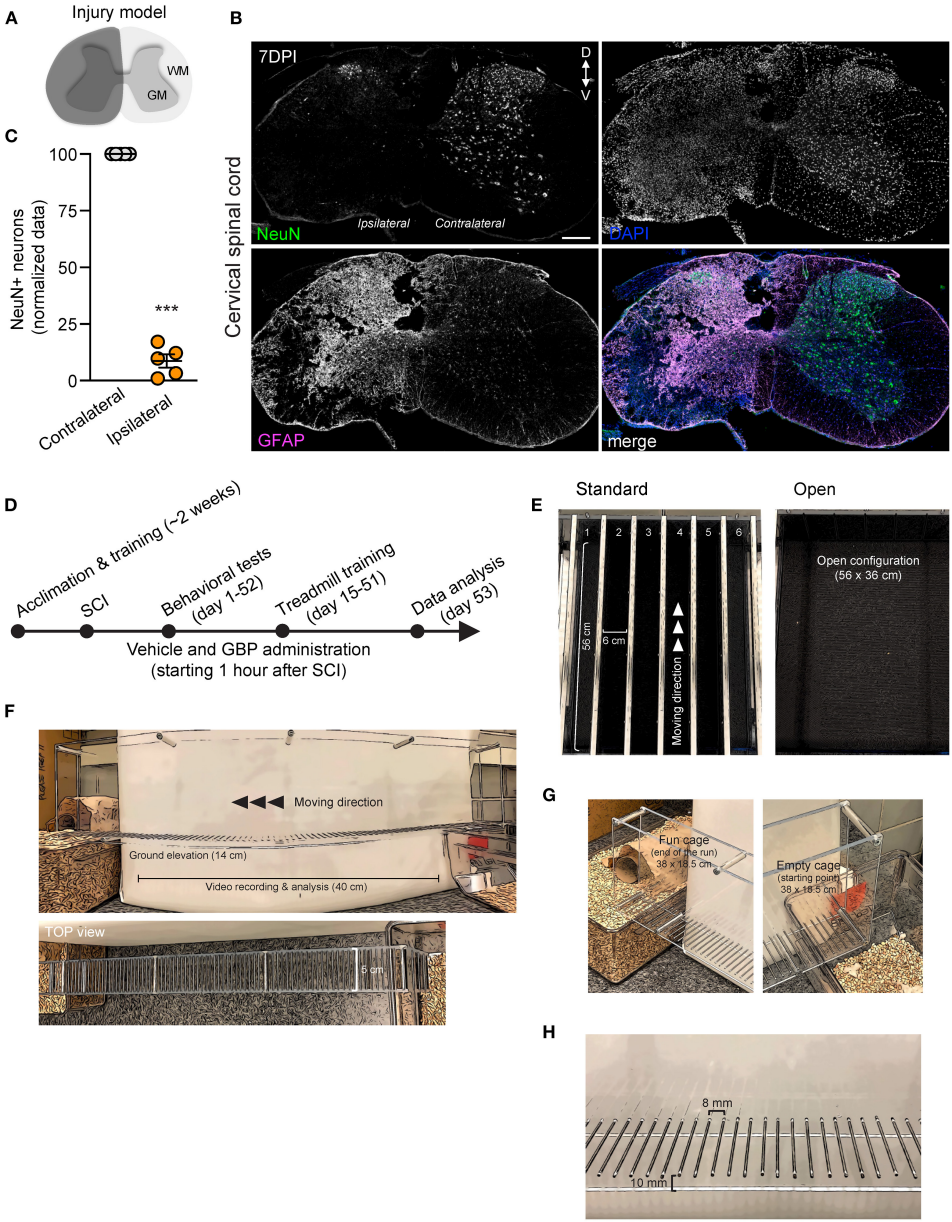
An experimental model of cervical SCI in adult mice and behavioral devices. **(A)** Schematic representation of C5 SCI experimental model. The region in dark gray represents the lesion site. **(B)** Coronal section of the injured spinal cord stained using GFAP and NeuN antibodies (DPI, days post-injury; D, dorsal; V, ventral). DAPI stained nuclei. Scale bar, 200 μm. **(C)** Quantification of **(B)**. Mean and SEM (paired 2-tailed Student's *t*-test ***p < 0.001, *n* = 5 biological replicates). **(D)** Experimental scheme. **(E)** Representative photographs of the treadmill apparatus in a standard and open (group runs) configuration. **(F–H)** Representative photographs of the skilled walking device. Mice were trained to cross the ladder from right (empty cage) to left (fun cage).

The treadmill apparatus (Columbus Instruments, Columbus, Ohio) consists of 6 individual lanes separated by solid gray walls of acrylic (length: 56 cm; height: 12.5 cm; width: 6 cm). See Figure 1E for a visual representation of the apparatus. In the original configuration, this setup allows for simultaneous training of up to 6 mice. A motor-driven rotor enables the experimenter to adjust the velocity of the belt (belt run: 44.5 cm). Prior to injury, all mice were acclimated to the treadmill apparatus for twelve consecutive days. Training sessions each lasted two and half hours and were conducted at the same time of day (12:00 p.m. EST−2:30 p.m. EST) to minimize the presence of confounding variables. During each session, mice were individually placed on the belt for the duration of 10–15 min and allowed to voluntarily participate. The speed of the treadmill was set at 6 m/min and slowly increased to 13 m/min over the course of the training period. Following injury, the mice were allotted a 14-day recovery period during which forelimb weight support and ground locomotion were regained. At 15 DPI, both GBP and control mice were reintroduced to the treadmill apparatus. Over the course of the following 4 months, all mice were engaged in an identical rehabilitation plan consisting of 58 training sessions. Sessions varied from 10 to 15 min in length, increasing over time. Given that mice were not forced onto the belt, participation was quantified by the percentage of time spent running on the belt during the exercise period. Established criteria consisted of the following: refusal (0), poor (0.33), moderate (0.67) or high (1) participation. For group runs (starting at 54 DPI, 2 times/week), the solid gray walls were removed.

For all behavioral tests described above, experimenters collecting and analyzing data were blinded to the treatment.

#### Horizontal Ladder Rung Walking Test

The horizontal ladder rung walking test apparatus consists of plexiglass side walls (length: 70 cm; height: 15 cm; lane width: 5 cm) and metal rungs (2 mm in diameter, 8 mm gap). See Figures 1F–H for a visual representation of the apparatus. Prior to injury, mice were trained to run in a unilateral direction (right to left) across the ladder from an unfamiliar, neutral cage (length: 38 cm; wide: 18.5 cm; height: 13 cm) to an enriched cage completely filled with bedding, caves and tunnels for play. For the duration of the entire study, a camera was positioned 33 cm away to consistently record each run (length analyzed: 40 cm). Upon review of video recordings, the percentage of correct forelimb steps was calculated. Quantification of correct paw placement was determined as described previously (Sun et al., 2020). Baseline values were collected and averaged for all mice prior to injury. Following injury, and at regular intervals until the study endpoint, mice were tested as described above. All videos were analyzed by blinded investigators.

#### Elevated Zero Maze

The maze (760369, Harvard Apparatus) consists of a cycling corridor (5.5 cm wide, 46 cm in diameter) elevated 54 cm above the floor. The maze is made of odor-resistant black perpex material with anti-reflective color and is divided into four quadrants of equal length with two opposing open quadrants and two opposing closed quadrants with black acrylic walls 15 cm in height. See **Figure 7F** for a visual representation of the apparatus. Under standard light conditions, a 5 min trial began with the animal placed at one of the intersections between the open and closed quadrants with the nose pointing to the closed quadrant. Between trials, the maze was cleaned with mild soap. A camera was positioned on top of the maze to consistently record each trial. Analysis of video recordings (e.g., total distance traveled, time spent in close and open arms etc.) was conducted using EthoVision XT software (Version 17, Noldus). To avoid habituation to the apparatus, no baseline values were collected.

#### Vertical Rearing

The mice were placed in a 500 ml clear beaker with a small amount of bedding on the bottom. The mice were allowed to move and explore independently. The number of vertical rearing episodes was counted over a 2 min period.

### RNA Extraction and Gene Expression Analysis

At 14 DPI, lesion sites (c.a. 1–1.3 mm thick coronal hemisections of the injured spinal cord) were dissected, flash-frozen and stored at−80°C. Tissues were homogenized and RNA was isolated using the Tri-Reagent (Sigma) and isopropanol protocol. RNA quality and integrity was confirmed by BioAnalyzer (Agilent). Gene expression was quantified using the nCounter NanoString (Genomics Core facility, The Ohio State University) neuroinflammation panel (770 genes). Technical normalization was performed to positive and negative controls and data were normalized using housekeeping genes (*Cskn2a2* and *Xpnpep1*) based on strong correlation with total counts (R^2^ > 0.8). Differential expression was analyzed using DESeq2 in R Studio. Results tables were generated based on injury (i.e., sham vs. SCI) and treatment (i.e., vehicle vs. GBP). The threshold for differentially expressed genes was set to *p* < 0.05. Ingenuity Pathway Analysis (IPA, QIAGEN) was used to identify the top 5 (up- and down-regulated) canonical pathways, master regulators and upstream regulators associated with the selected genes. IPA results are presented by z-score.

The same RNA samples used for Nanostring were used for cDNA synthesis (500 ng of RNA) using random hexamers from the SuperScript VILO cDNA synthesis kit (11754050, Thermo Fisher Scientific). The cDNA was then used in a real-time PCR (QuantStudio 3, Applied Biosystem) using Fast SYBR Green Master Mix (4385612, Applied Biosystem). Melting curve reactions were run with each primer set. The β-actin gene was used for normalization. The sequences of the primers used were as follows:

IL-4_s 5′-AGATGGATGTGCCAAACGTCCTCA-3′

IL-4_as 5′-AATATGCGAAGCACCTTGGAAGCC-3′

IL1b_s 5′-TGCCACCTTTTGACAGTGATG-3′

IL1b_as 5′-TGATGTGCTGCTGCGAGATTT-3′

ITGAM_s 5′-TGGCCTATACAAGCTTGGCTTT-3′

ITGAM_as 5′-AAAGGCCGTTACTGAGGTGG-3′

IL6_s 5′-TCTATACCACTTCACAAGTCGGA-3′

IL6_as 5′-GAATTGCCATTGCACAACTCTTT-3′

TNFα_s 5′-GATCGGTCCCCAAAGGGATG-3′

TNFα_as 5′-TGTGAGGGTCTGGGCCATAG-3′

IL10_s 5′-ATTTGAATTCCCTGGGTGAGAAG-3′

IL10_as 5′-CACAGGGGAGAAATCGATGACA-3′

IL1α_s 5′-CGCTTGAGTCGGCAAAGAAAT-3′

IL1α_as 5′-CTTCCCGTTGCTTGACGTTG-3′

CD68_s 5′-ACTGGTGTAGCCTAGCTGGT-3′

CD68_as 5′-CCTTGGGCTATAAGCGGTCC-3′

C3_s 5′-AGCTTCAGGGTCCCAGCTAC-3′

C3_as 5′-GCTGGAATCTTGATGGAGACGC-3′

C1qA_s 5′-TCCAGTTTGATCGGACCACG-3′

C1qA_as 5′-GGATTTCCTGGAGCCCCATC-3′

F4/80_s 5′-TGCATCTAGCAATGGACAGC-3′

F4/80_as 5′-GCCTTCTGGATCCATTTGAA-3′

β-actin_s 5′-ACAGCTTCACCACCACAGCTGA-3′

β-actin_as 5′-GAGGTCTTTACGGATGTCAACGTC-3′.

Normalized expression was calculated as dCt (gene norm) =

Ct gene of interest – Ct β-actin and normalized expression =

2-dCt (gene norm).

### BrdU Incorporation

At 14 DPI, SCI mice administered vehicle and GBP received intraperitoneal BrdU (150 mg/kg body weight, dissolved at 15 mg/ml in 0.9% saline, Sigma) injections. BrdU injections were spaced 2 h apart and mice were perfused 2 h after the third and last injection.

### Statistical Model and Analysis

According to the nature of the experimental variables we intended to study, we attributed a binomial distribution to the behavioral response so that each behavioral measurement was considered as the probability of success. For the horizontal ladder rung walking test, this indicated the probability of correct placement. For the degree of participation in the treadmill training, it referred to the probability that a mouse will reach the training platform (i.e., the running belt) after being loaded on the stationary docking station. By representing the experimental data in this fashion, we can begin to understand the relationship between experimental variables in a probabilistic rather than a deterministic model like linear model fitting. In this study, we applied a generalized logistic linear model with the Logit function in the calculation of restricted maximum likelihood. The data sets contained repeated measures that required the use of a mixed effect model to control the variable coefficients for random intercept/slope in each subject. The glmer function in “lme4” package of R enabled us to model the effect of time and experimental variables and their interaction while controlling each estimate for the random effect of subjects.

**Figure 2:**
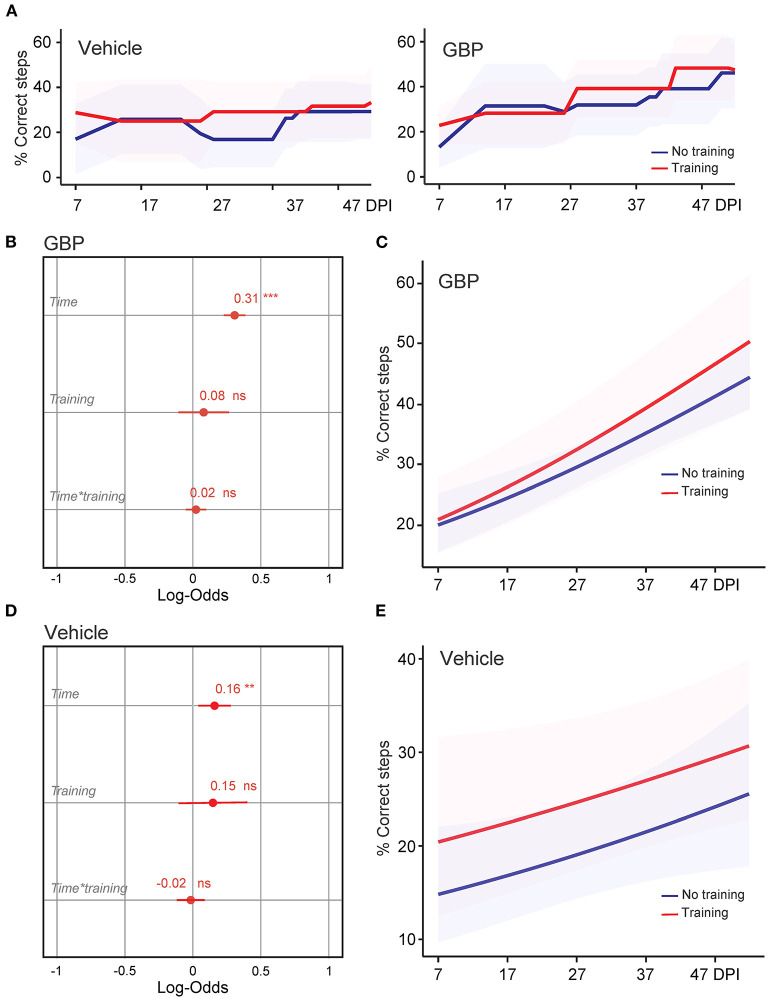
Treadmill training in combination with GBP administration does not improve skilled walking. **(A)** Recovery of forelimb skilled locomotor function was measured using the horizontal ladder rung-walking test. Mean and standard deviation (vehicle without treadmill training *n* = 25, vehicle with treadmill training *n* = 13, GBP without treadmill training *n* = 25, GBP with treadmill training *n* = 14). Of note, SCI mice (vehicle and GBP) without treadmill training originated from our previous study (Sun et al., 2020). **(B)** Analysis of **(A)**. Variable effects on forelimb skilled locomotor function in mice administered GBP are displayed as coefficients with confidence intervals (Generalized linear mixed effect model for repeated measures ***p < 0.001, ns not significant, GBP without treadmill training *n* = 25 and GBP with treadmill training *n* = 14 mice). Of note, SCI mice administered GBP without treadmill training originated from our previous study (Sun et al., 2020). **(C)** Predicted changes in forelimb skilled locomotor function in mice administered GBP. Shaded error bands indicate confidence intervals. **(D)** Analysis of **(A)**. Variable effects on forelimb skilled locomotor function in mice administered vehicle are displayed as coefficients with confidence intervals (Generalized linear mixed effect model for repeated measures **p < 0.01, ns not significant, vehicle without treadmill training *n* = 25 and vehicle with treadmill training *n* = 13 mice). Of note, SCI mice administered vehicle without treadmill training originated from our previous study (Sun et al., 2020). **(E)** Predicted changes in forelimb skilled locomotor function in mice administered vehicle. Shaded error bands indicate confidence intervals.

**Figure 3:**
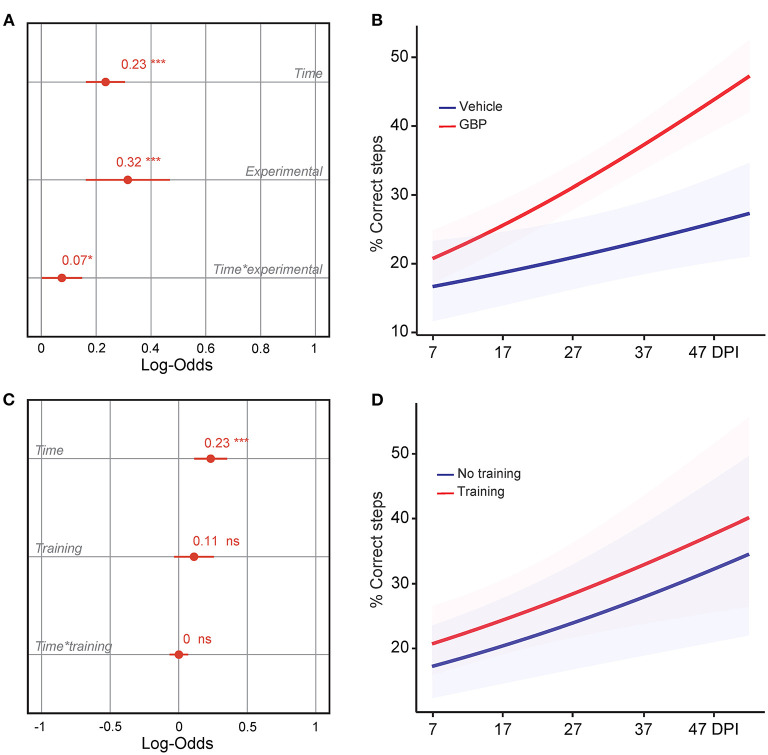
Mice administered GBP are more likely to regain forelimb function after SCI, independent of treadmill training. **(A)** Variable effects on forelimb skilled locomotor function in mice administered vehicle or GBP are displayed as coefficients with confidence intervals (Generalized linear mixed effect model for repeated measures *p < 0.05, ***p < 0.001, vehicle *n* = 38 and GBP *n* = 39 mice). Of note, SCI mice (vehicle and GBP) without treadmill training originated from our previous study (Sun et al., 2020). **(B)** Predicted changes in forelimb skilled locomotor function in SCI mice administered vehicle or GBP. Shaded error bands indicate confidence intervals. **(C)** Variable effects on forelimb skilled locomotor function in SCI mice with or without treadmill training are displayed as coefficients with confidence intervals (Generalized linear mixed effect model for repeated measures ***p < 0.001, ns not significant, mice without treadmill training *n* = 50 and mice with treadmill training *n* = 27 mice). Of note, SCI mice without treadmill training originated from our previous study (Sun et al., 2020). **(D)** Predicted changes in forelimb skilled locomotor function in SCI mice with or without treadmill training. Shaded error bands indicate confidence intervals.

**Figure 4:**
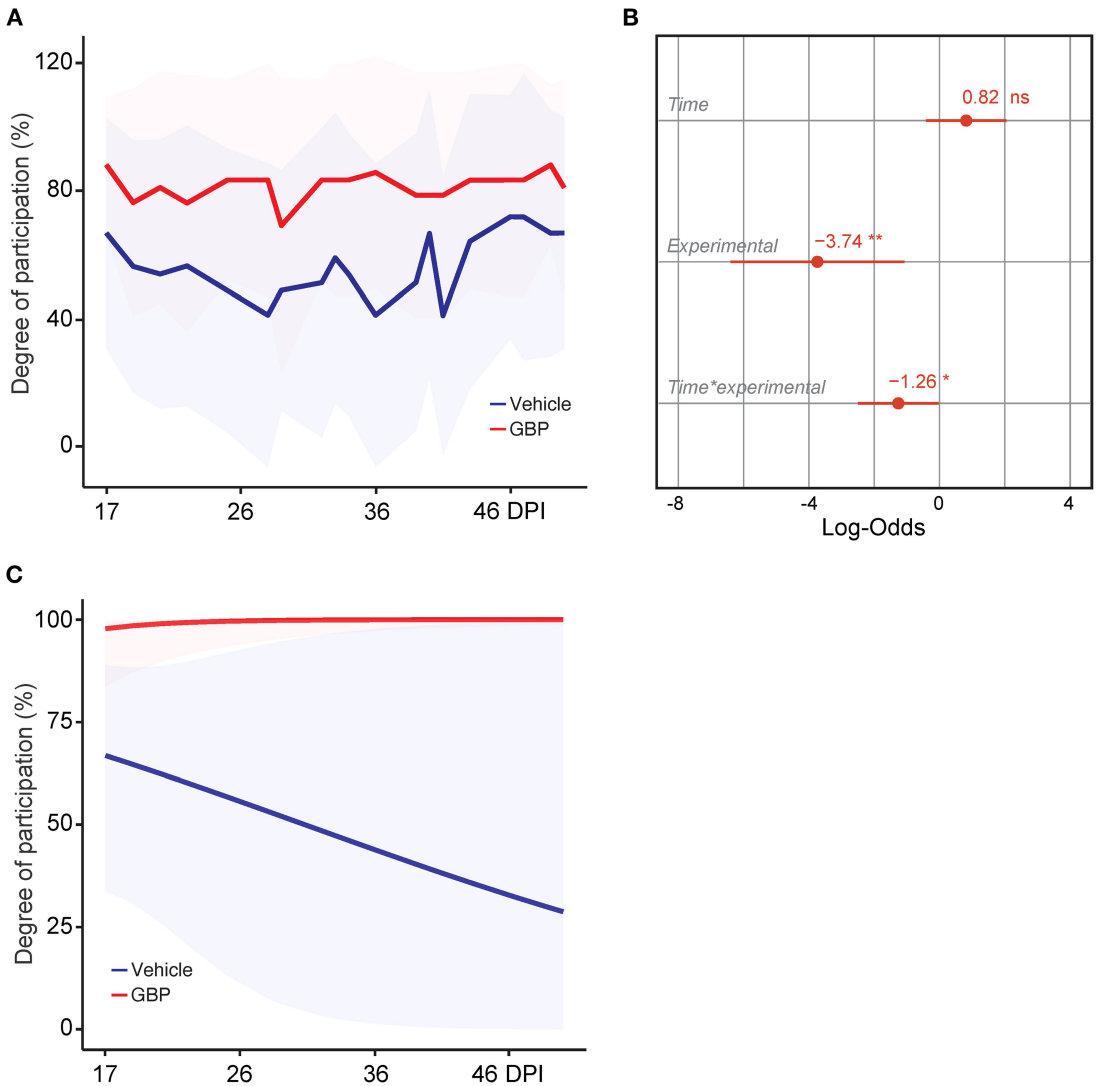
Control mice lack motivation after chronic SCI. **(A)** Intrinsic motivation was measured by assessing the degree of participation in rehabilitative treadmill training. Mean and standard deviation (vehicle with treadmill training *n* = 13 and GBP with treadmill training *n* = 14). **(B)** Analysis of **(A)**. Variable effects on motivation toward rehabilitative treadmill training in SCI mice administered vehicle and GBP are displayed as coefficients with confidence intervals (Generalized linear mixed effect model for repeated measures *p < 0.05, **p < 0.01, ns not significant, vehicle with treadmill training *n* = 13 and GBP with treadmill training *n* = 14 mice). **(C)** Predicted changes in motivation toward the rehabilitative treadmill training in SCI mice administered vehicle or GBP. Shaded error bands indicate confidence intervals.

Statistical analysis was performed using R (v. 4.1.1; R Core Team) as follows: generalized linear mixed effects model for repeated measures (Figures 2C,E, 3A,C, 4B, **F8">8B,E**, **9B,E**). Applied R packages include lme4 (version 1.1-27.1) where glmer function was used for fitting generalized linear mixed effects models (Bates et al., 2015) (Figures 2C–F, 3A–D, 4B,C, **8B,C,E,F**, **9B–E**), ggeffects (version 1.1.1) where ggpredict function was used to generate predictions (Lüdecke, 2018) (Figures 2D,F, 3B,D, 4C, **8C,F**, **9C,D**), sjPlot (version 2.8.10) where plot_model function was used to plot fixed and random effects (Lüdecke, 2021) (Figures 2C,E, 3A,C, 4B, **8B,E**, **9B,E**), ggplot2 where ggplot function was used to plot results (Wickham, 2016) (Figures 2B,D,F, 3B,D, 4C, **8A,C,D,F**, **9A,C,D**). Statistical analysis was also performed using Prism (version 9.3.1; GraphPad Software) as follows: Wilcoxon rank sum test (**Figures 6C**, **7B,F,I,J**). For all analyses performed, significance was defined as ^*^p < 0.05, ^**^p < 0.01 and ^***^p < 0.001. Exact values of *n* and definition of measures are shown in the corresponding figure legends.

### Study Approval

All animal experiments were performed in accordance with and with the approval of the Institutional Animal Care and Use Committee at The Ohio State University.

## Results

### Treadmill Training in Combination With GBP Administration Does Not Improve Skilled Walking

We recently demonstrated that mice administered GBP recovered upper extremity function following cervical SCI (Sun et al., 2020). By pharmacologically blocking Alpha2delta1/2 subunits of voltage-gated calcium channels (Gee et al., 1996; Gong et al., 2001), gabapentinoids augment mechanisms of structural plasticity and facilitate the regeneration of ascending sensory and descending motor pathways following SCI and ischemic stroke in adult mice (Tedeschi et al., 2016, 2022; Sun et al., 2020). Given that cardiovascular exercise strategies, including treadmill training, have been proven to enhance neuronal plasticity and promote motor recovery (Jo and Perez, 2020; Faw et al., 2021), we hypothesized that the combination of an aerobic-based rehabilitation regimen with GBP administration will maximize recovery of forelimb function in mice following cervical SCI by strengthening synaptic connections along the sensorimotor axis. To test this hypothesis, adult mice sustained a cervical (C)5 injury to the spinal cord (Figures 1A–C), yielding significant impairments to forelimb function on the left side. To assess the effectiveness of cardiovascular exercise in the context of neurological recovery, mice were subjected to a voluntary treadmill-based rehabilitation protocol (Figure 1D). Prior to injury, all mice were acclimated to the treadmill apparatus (Figure 1E) for 12 consecutive days. Beginning 1 h after SCI, and until the end of the study, mice were administered daily injections of either GBP (46 mg/kg in body weight) or vehicle (0.9% saline, also referred as control) (Figure 1D). Following SCI, mice were allowed to recover forelimb weight support and ground locomotion. Both GBP and control mice were then reintroduced to the treadmill apparatus at 15 days post injury (DPI). Over the course of the following 5 weeks, all mice were engaged in an identical rehabilitation plan consisting of 20 training sessions (3–5 sessions/week; 10–15 min/session). At multiple time points throughout the entire rehabilitation period, recovery of skilled walking was assessed using the horizontal ladder rung walking test (Metz and Whishaw, 2009) (Figures 1F–H). Following data collection, the results of the current study were directly compared to those of two of our previously published data sets (Sun et al., 2020) (Figure 2A). These published data sets originated from two independent cohorts of mice subjected to the same experimental conditions as described above, with the exception of the treadmill training. Interestingly, our findings indicate that no additive, beneficial effect of voluntary treadmill training is observed in mice administered GBP in the context of increased forelimb recovery (Figure 2B). Nonetheless, mice administered GBP consistently recovered forelimb function over the course of weeks following SCI (Figures 2B,C), as we previously reported (Sun et al., 2020). Unexpectedly, however, we also failed to find an additive effect of voluntary treadmill training in the context of increased forelimb recovery in mice administered vehicle (Figures 2D,E). Even so, a certain degree of spontaneous recovery in forelimb function was still observed in these mice over the course of weeks following SCI. We validated these conclusions by further interrogating our data sets. After merging all behavioral data from the independent cohorts of mice and controlling for the presence or absence of treadmill training, we then asked which experimental condition is more subjected to changes in terms of enhanced forelimb performance on the horizontal ladder rung walking test. As expected, the model indicated that mice administered GBP are more likely to regain forelimb function over the course of days and weeks after SCI, regardless of whether or not they were subjected to the training protocol (Figures 3A,B). Subsequently, we asked to what extent does voluntary treadmill training impact the recovery of forelimb function. After controlling for the presence or absence of GBP administration, the model suggested that voluntary treadmill training is not a strong predictor of successful recovery as it does not impact recovery of forelimb function after a severe cervical SCI (Figures 3C,D). Instead, time had a significant effect on recovery of forelimb function in the acute and subacute phases following SCI (Figures 3C,D). Together, these data show that GBP administration and voluntary treadmill training, when combined, do not maximize recovery of forelimb function after SCI.

### A Lack of Engagement Within the Training Protocol Is Observed in SCI Mice Administered Vehicle

Motivation plays a crucial role in rehabilitative interventions and is thereby often used to determine rehabilitation outcome (Colombo et al., 2007). In consideration of this fact, we scored active engagement toward the voluntary treadmill-based rehabilitation protocol by calculating the percentage of time spent running on the belt during the exercise period. Results indicate that mice administered GBP were consistently engaged in the rehabilitation protocol (Figure 4A). Interestingly, control (i.e., vehicle) mice exhibited lower participation when compared to mice administered GBP (Figures 4A,B). Moreover, the model predicted a steep decline in participation toward the rehabilitation protocol as control mice entered the chronic phase of SCI (Figure 4C). Whereas mice administered GBP continued to recover function into the chronic phase (i.e., >28 DPI), little to no additional recovery was observed during these time points in control mice (Figure 2A). Up until this point, participation within the mice was based solely on intrinsic motivation. As numbers began to plateau, participation via intrinsic motivation was maximized. Manipulating environmental/extrinsic factors may be sufficient to rescue active engagement in the training protocol and therefore maximize recovery, especially at chronic time points.

### GBP Administration Does Not Attenuate Neuroinflammatory Gene Expression

**Figure 5:**
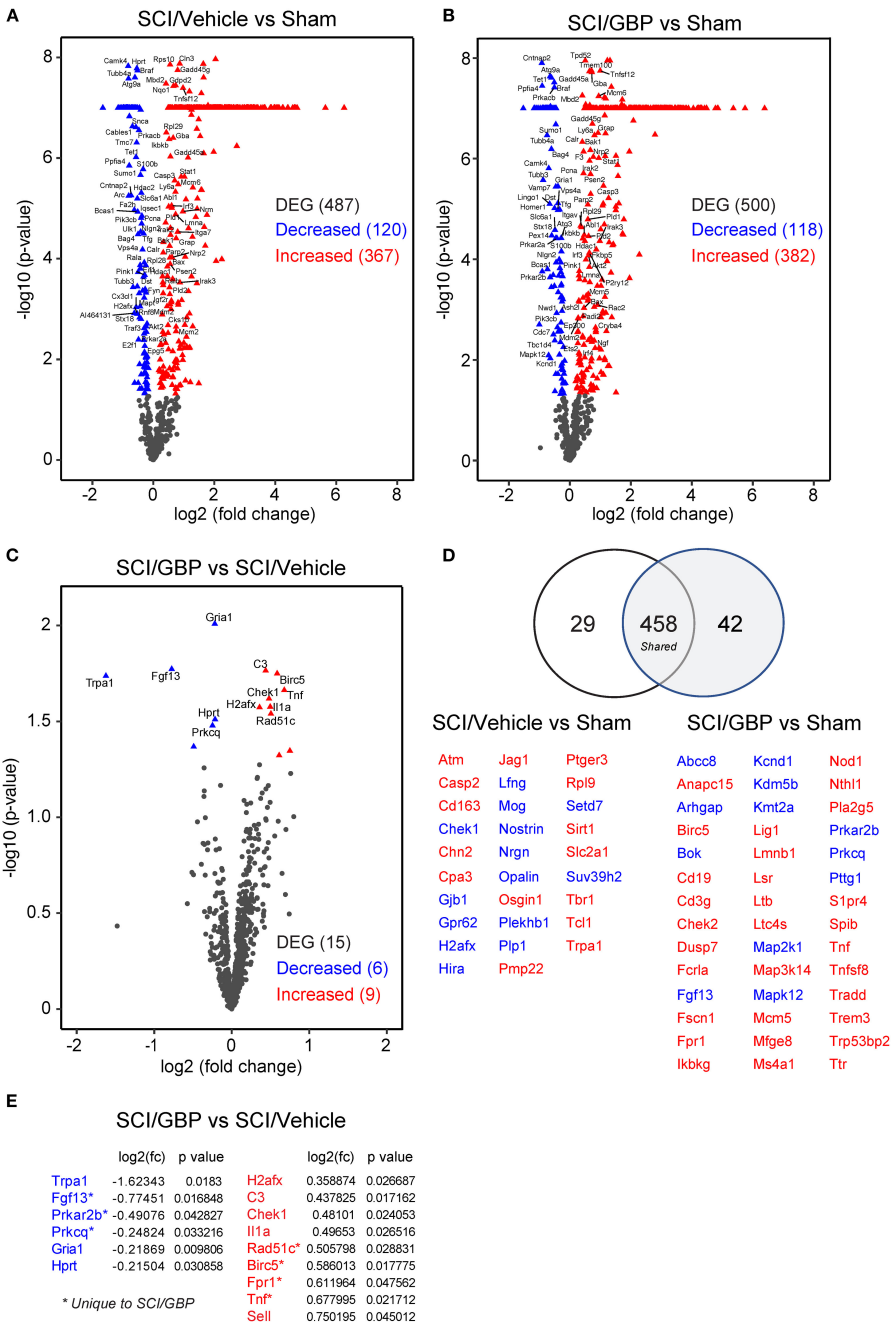
GBP administration does not attenuate neuroinflammatory gene expression. **(A–C)** Volcano plots of NanoString neuroinflammatory panel. The blue and red triangles represent differentially expressed genes (DEG). **(D)** The number of DEG in SCI mice administered vehicle and GBP are represented in the Venn diagram. In blue and red are the DEG associated to SCI/Vehicle and SCI/GBP groups. **(E)** DEG enriched in the SCI/GBP over the SCI/Vehicle group (*p* < 0.05).

Accumulating evidence indicates that neuroinflammation may play a role in the deterioration of mental health following SCI (Allison and Ditor, 2015; Brakel et al., 2021). To assess if GBP administration attenuates neuroinflammatory gene expression profiles, we extracted RNA from the lesion site at 14 DPI and ran a NanoString neuroinflammatory panel of 770 genes. RNA samples originating from sham-operated mice served as a reference for normalization of SCI-dependent gene changes. Whereas SCI profoundly impacted gene expression (Figures 5A,B), no major changes were found when comparing neuroinflammatory gene signatures in SCI/GBP and SCI/vehicle groups (Figures 5C,D). There were 15 genes related to neuroinflammation that were differentially expressed at 14 DPI in mice administered GBP. Of these, 6 (*Trpa1, Fgf13, Prkar2b, Prkcq, Gria1, Hprt*) were attenuated and 9 (*H2afx, C3, Chek1, Il1a, Rad51c, Birc5, Fpr1, Tnf*, *Sell*) were enhanced at the lesion site (Figure 5E). Of the attenuated genes in SCI mice administered GBP, *Trpa1* encodes the transient receptor potential ankyrin 1 (TRPA1), a non-selective cation channel expressed in sensory pathways. TRPA1 is activated during hypoxia (Chen et al., 2020) and activation of Trpa1-positive sensory pathways has been shown to induce hypothermia (Matsuo et al., 2021). *Fgf13* encodes the fibroblast growth factor 13 (FGF13), a member of the FGF family that is involved in a variety of biological processes including development, cell growth, morphogenesis and invasion. Loss of *Fgf13* in murine sensory neurons selectively abolishes heat nociception (Yang et al., 2017). *Gria1* encodes the glutamate ionotropic receptor AMPA type subunit 1 (GRIA1), a member of the family of AMPA receptors associated with excitatory glutamatergic transmission. Alteration of glutamate receptor signaling contributes to glutamate-mediated excitotoxicity after SCI (Li and Stys, 2000; Dong et al., 2009). Of the enhanced genes in mice administered GBP, *Birc5* encodes the baculoviral IAP repeat containing 5 (also known as Survivin), a member of the inhibitor of apoptosis gene family. Survivin silencing has been shown to inhibit the proliferation and differentiation of neural precursor cells in the dentate gyrus of the hippocampus after brain trauma, leading to poor functional recovery (Zhang et al., 2015). *Fpr1* encodes the formylpeptide receptor 1 (FPR1), a member of the family of G protein-coupled chemoattractant receptors. Formylpeptide receptors promote the migration and differentiation of neural stem cells in mice and rats (Wang et al., 2016; Zhang et al., 2017).

**Figure 6:**
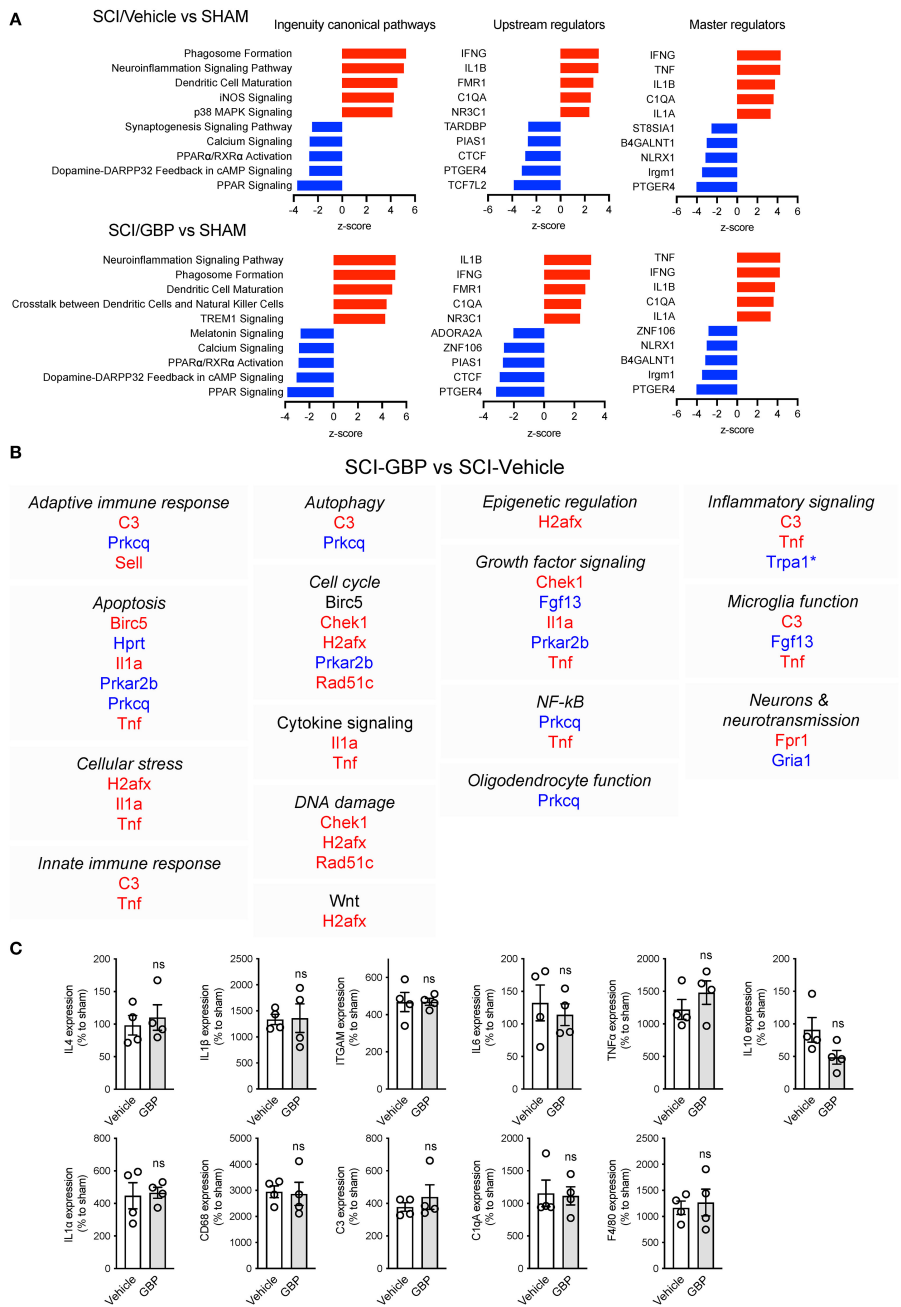
**(A)** Ingenuity analysis of canonical pathways (z-score, *p* < 0.05), upstream (z-score, *p* < 0.05) and master regulators (z-score, *p* < 0.05) in SCI/Vehicle and SCI/GBP groups. **(B)** List of GBP-dependent changes in gene expression and associated cellular function. **(C)** Gene expression of inflammatory markers was analyzed by qRT-PCR. Mean and SEM (Wilcoxon rank sum test, ns: not significant; Sham *n* = 4, SCI/Vehicle *n* = 4, SCI/GBP *n* = 4).

IPA of canonical pathways, upstream and master regulators showed high similarity between vehicle and GBP-mediated neuroinflammatory gene profiles at 14 DPI when compared to sham (Figure 6A). Analysis of the GBP-dependent changes on gene expression after SCI indicated a number of cellular functions including apoptosis, cell cycle, growth factor and inflammatory signaling may be impacted (Figure 6B).

Next, we subjected the same RNA samples to qPCR analysis. At 14 DPI, the expression profiles of inflammatory markers including *Il4, Il1*β, *ITGAM, Il6, TNF*α*, Il10, Il1*α*, CD68, C3, C1qA, and F4/80* were comparable in mice administered vehicle and GBP (Figure 6C). Collectively, these data indicate subtle changes in GBP-dependent regulation of neuroinflammatory gene expression after SCI.

### GBP Administration Promotes Hippocampal Neurogenesis and Counteracts Anxiety-Like Behavior After SCI

**Figure 7:**
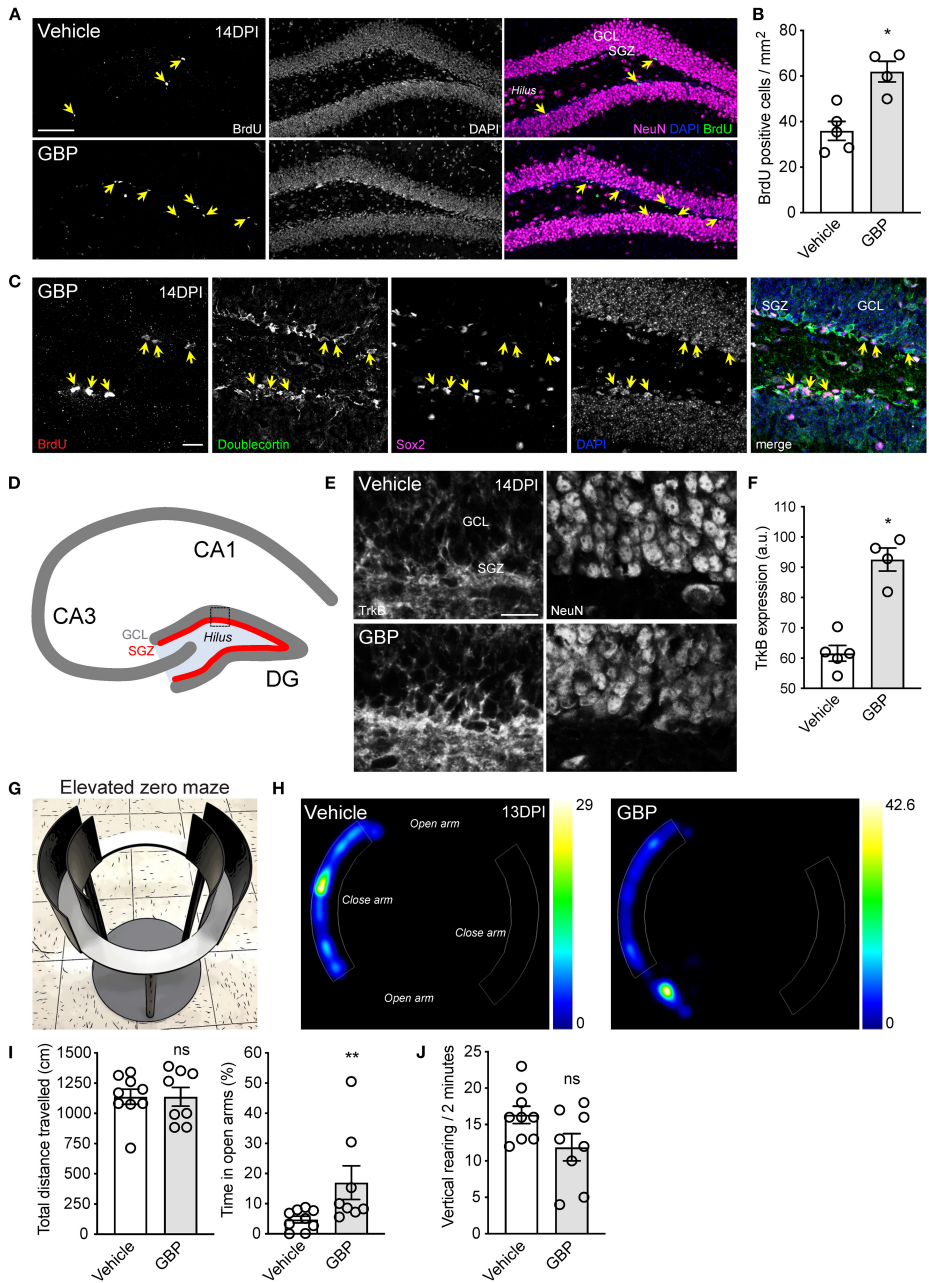
Chronic administration of GBP promotes hippocampal neurogenesis and counteracts anxiety-like behavior. **(A)** Representative fluorescence images of hippocampal sections from adult mice 14 DPI. Yellow arrows indicate BrdU positive cells in the subgranular zone (SGZ) of the dentate gyrus (GCL: granule cell layer). Scale bar, 100 μm. **(B)** Quantification of **(A)**. Mean and SEM (Wilcoxon rank sum test, *p < 0.05, vehicle *n* = 5 and GBP *n* = 4). **(C)** Representative fluorescence images of BrdU positive (yellow arrows) neural progenitor cells and immature neurons in the subgranular zone of the dentate gyrus. Scale bar, 20 μm. **(D)** Schematic representation of the murine hippocampus. **(E)** Representative fluorescence images of the dentate gyrus from SCI mice administered vehicle and GBP. Scale bar, 20 μm. **(F)** Quantification of **(E)**. Mean and SEM (Wilcoxon rank sum test, *p < 0.05, vehicle *n* = 5 and GBP *n* = 4). **(G)** Representative photograph of the elevated zero maze used to assess anxiety-like behavior in mice. **(H)** Representative heat map tracing of the elevated zero maze at 13 DPI for SCI mice administered vehicle and GBP. **(I)** Quantification of **(H)**. Mean and SEM (Wilcoxon rank sum test, ns not significant, *p < 0.05, vehicle *n* = 9 and GBP *n* = 8). **(J)** Quantification of vertical rearing at 13 DPI. Mean and SEM (Wilcoxon rank sum test, ns not significant, vehicle *n* = 9 and GBP *n* = 8).

New neurons are generated from dividing progenitor cells in the subgranular zone of the dentate gyrus of the adult mammalian hippocampus (Eriksson et al., 1998; Sorrells et al., 2018). Impairment of adult hippocampal neurogenesis is associated with mental deterioration and anxiety-related behaviors (Revest et al., 2009). Not only does SCI impair sensory and motor functions, it also negatively impacts psychological wellbeing (Krause et al., 2000; Brakel et al., 2019). Adult hippocampal neurogenesis can be stimulated by chronic administration of antidepressants and by voluntary exercise (Malberg et al., 2000; van Praag et al., 2005; Perera et al., 2007). Given that mice administered GBP displayed a better recovery profile and were consistently engaged in the rehabilitation protocol, we questioned whether or not GBP administration increases hippocampal neurogenesis after SCI. To answer this question, adult mice sustained a C5 SCI (Figures 1A–C). Beginning 1 h after SCI, and until the end of the study, mice were administered daily injections of either GBP (46 mg/kg in body weight) or vehicle (0.9% saline). Of note, these mice were not subjected to voluntary treadmill training. At 14 DPI, we injected bromodeoxyuridine (BrdU) to label proliferating cells in the subgranular zone of the dentate gyrus of the hippocampus. Histological analysis confirmed SCI mice administered GBP had enhanced hippocampal neurogenesis as shown by the increase in the number of BrdU positive cells representing neural progenitor cells and immature neurons in the subgranular zone (Figures 7A–C). TrkB plays a crucial role in hippocampal neurogenesis (Li et al., 2008). Hence, we asked if increased neurogenesis in mice administered GBP is associated with changes in TrkB expression. Strikingly, we found a ~50% increase in TrkB expression in the subgranular zone of the dentate gyrus in mice administered GBP at 14 DPI (Figures 7D–F). We also discovered that mice administered GBP had less anxiety-like behavior in the elevated zero maze compared to vehicle control at 13 DPI (Figures 7G–I). At the same time point, there was a non-significant downtrend in the number of vertical rearing episodes in mice administered GBP (Figure 7J). Taken together, our data indicate that chronic administration of GBP after SCI stimulates hippocampal neurogenesis and TrkB expression in the subgranular zone of the dentate gyrus, likely contributing to suppression of anxiety-like behavior.

### An External, Social Motivator Rescues Participation in SCI Mice Administered Vehicle, Even in the Chronic Phase

**Figure 8:**
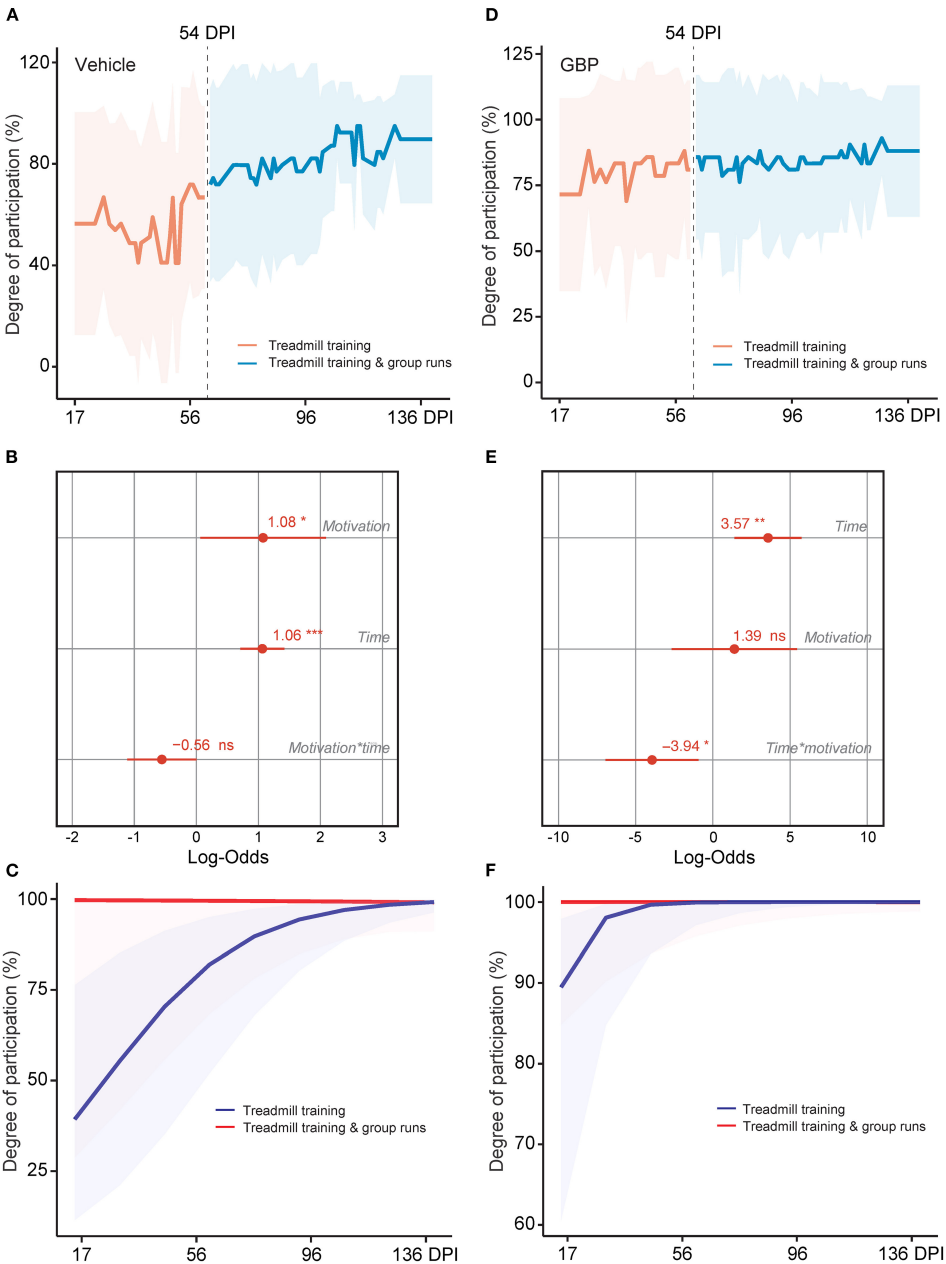
A social motivator rescues participation in control mice, even at chronic time points. **(A)** Change in motivation in control mice was measured after the addition of a social motivator (e.g., group runs) into the treadmill training starting at 54 DPI. Mean and standard deviation (vehicle with treadmill training *n* = 13). **(B)** Analysis of **(A)**. Variable effects on motivation toward rehabilitative treadmill training in SCI mice administered vehicle are displayed as coefficients with confidence intervals (Generalized linear mixed effect model for repeated measures *p < 0.05, ***p < 0.001, ns not significant, vehicle with treadmill training *n* = 13). **(C)** Predicted changes in motivation toward the rehabilitative treadmill training in SCI mice administered vehicle. Shaded error bands indicate confidence intervals. **(D)** Change in motivation in mice administered GBP was measured after addition of a social motivator (e.g., group runs) into the treadmill training starting at 54 DPI. Mean and standard deviation (GBP with treadmill training *n* = 14). **(E)** Analysis of **(D)**. Variable effects on motivation toward rehabilitative treadmill training in SCI mice administered GBP are displayed as coefficients with confidence intervals (Generalized linear mixed effect model for repeated measures *p < 0.05, **p < 0.01, ns not significant, GBP with treadmill training *n* = 14). **(F)** Predicted changes in motivation toward the rehabilitative treadmill training in SCI mice administered GBP. Shaded error bands indicate confidence intervals.

It is well established that the degree of difficulty and the duration of the exercise directly influences motivation (Duncan et al., 2010). In addition, social factors as well as the quality and quantity of feedbacks presented to the participant also influence motivation and therefore rehabilitation outcome. In SCI mice administered vehicle, the potential for intrinsic motivation was drained at chronic time points. Hence, we asked whether or not external motivating stimuli would be sufficient to re-engage chronic SCI mice into voluntary rehabilitative training. During the horizontal ladder rung walking test, mice were required to cross in a unilateral direction from an empty cage to an enriched cage filled with bedding, caves, tunnels and their cagemates (Figures 1F–H). As the duration of the test was short (e.g., 3–5 s/run) and extrinsic motivators were provided, 100% of the mice in both experimental groups completed this task at acute and chronic time points (vehicle with treadmill training *n* = 13 and GBP with treadmill training *n* = 14). In the case of the treadmill training, this task requires not only technical skill (e.g., forelimb-hindlimb coordination, high speed locomotion), as in the horizontal ladder rung walking test, but also endurance (e.g., muscle strength) to successfully complete the 10–15 min of continuous exercise. Thus, the external motivator must be strong enough to overcome the intrinsic, mental barriers facing the mice throughout the training period. Mice, much like humans, are social creatures by nature. Thus, subjecting them to the treadmill apparatus may be stressful not only due to the intensity of the exercise, but also due to the isolation of the animal over an extended period of time (Figure 1E). Starting at 54 DPI, we implemented a group run strategy (Supplementary Video 1). Instead of letting the animals run alone, we allowed them to run together, while still assessing them on an individual basis. Following the initiation of the group run strategy, an immediate increase in participation toward the voluntary treadmill training was observed in mice administered vehicle (Figures 8A,B). Importantly, our analysis indicates that the increase in participation is not the result of time (Figure 8B). While a plateau was expected as animals were already in the chronic phase of SCI, participation continued to increase in mice administered vehicle, with maximal participation observed at the end of the study (i.e., 4 months after SCI) (Figure 8A). Mice administered GBP remained consistently engaged in the rehabilitation protocol, independent of the initiation of the group run strategy (Figures 8D–F). These data indicate that chronic administration of gabapentinoids may counteract diminished motivation as the result of deterioration of mental health following SCI, especially at chronic time points. In addition, our results suggest that an external, social motivator effectively rescues participation in control mice, even in the chronic phase of SCI.

### Overcoming Motivational Barriers During Rehabilitative Training Promotes Recovery After SCI

**Figure 9:**
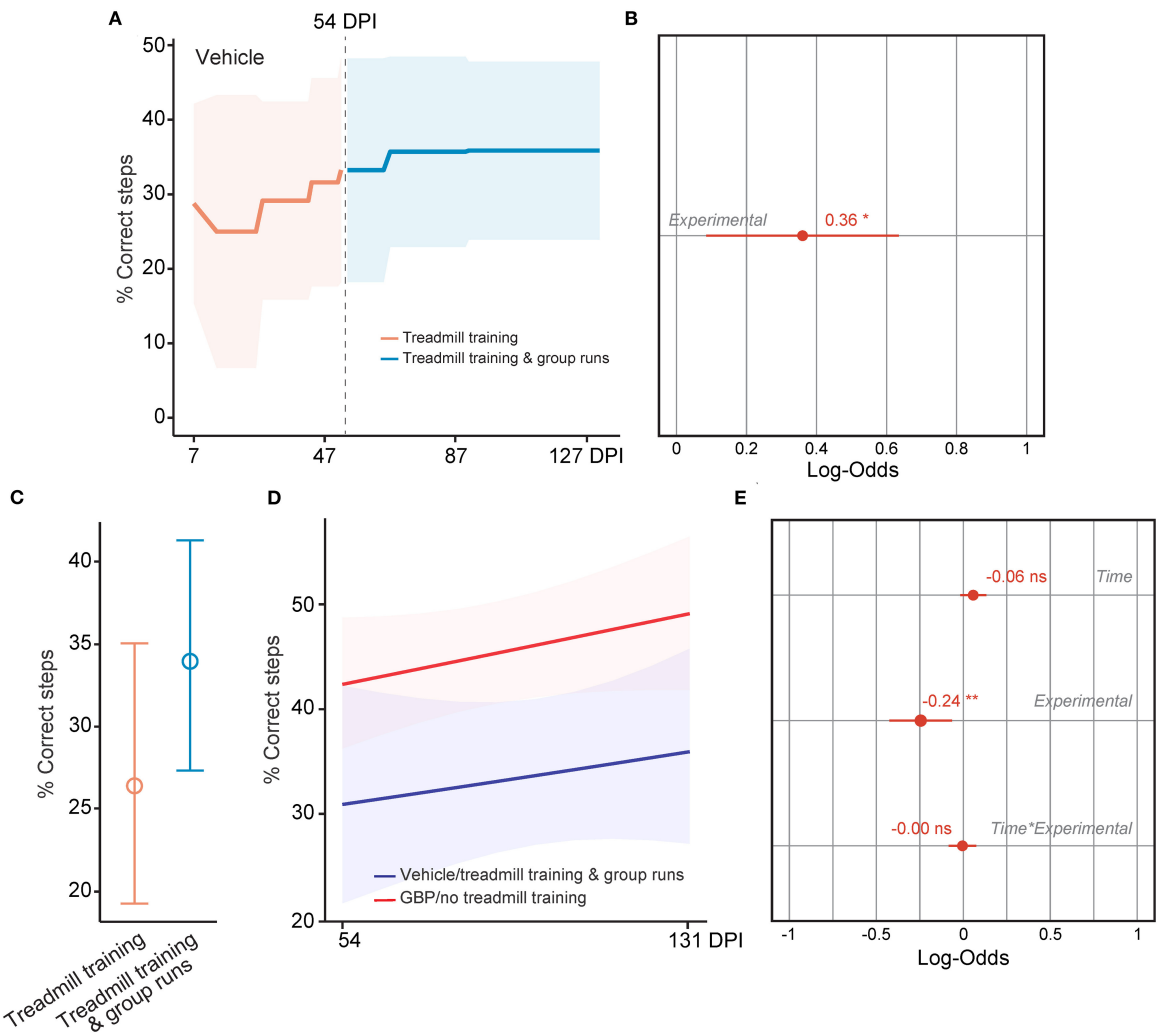
Rescuing motivation promotes forelimb recovery in chronic SCI mice. **(A)** Recovery of forelimb skilled locomotor function in control mice was measured using the horizontal ladder rung-walking test. Mean and standard deviation (vehicle with treadmill training *n* = 13). **(B)** Analysis of **(A)**. Variable effect (Variable ~ 0 or pre vs. 1 or post 54 DPI) on forelimb skilled walking in SCI mice administered vehicle is displayed as coefficient with confidence intervals (Generalized linear mixed effect model for repeated measures *p < 0.05, vehicle with treadmill training *n* = 13). **(C)** Predicted changes in forelimb skilled locomotor function in SCI mice administered vehicle before and after implementation of group runs during treadmill training. Mean (value) and confidence intervals. **(D)** Predicted changes in forelimb skilled locomotor function in SCI mice administered vehicle after implementation of group runs during treadmill training or SCI mice administered GBP alone. Shaded error bands indicate confidence intervals. **(E)** Analysis of **(D)**. Variable effects on forelimb skilled locomotor function in SCI mice administered vehicle after implementation of group runs during treadmill training or SCI mice administered GBP alone are displayed as coefficients with confidence intervals (Generalized linear mixed effect model for repeated measures **p < 0.01, ns not significant, vehicle *n* = 13 and GBP *n* = 25 mice). Of note, SCI mice administered GBP without treadmill training and group runs originated from our previous study (Sun et al., 2020).

Lastly, we tested if mice administered vehicle displayed increased forelimb function at chronic time points after SCI as they re-engaged in the voluntary treadmill training protocol following the introduction of the social motivator. As described above, we assessed skilled walking in these mice using the horizontal ladder rung walking test. To our surprise, we found that the behavioral performance in control mice increased after 54 DPI as shown by the increase in the number of correct steps following implementation of group runs in the treadmill training protocol (Figures 9A–C). When directly compared, however, daily administration of GBP alone is superior in promoting neurological recovery after SCI (Figures 9D,E), underscoring the importance of creating favorable conditions for SCI repair before entering the chronic phase. Collectively, these data provide strong evidence that breaking mental barriers may boost spontaneous recovery, even in the chronic phase of SCI.

## Discussion

Administration of gabapentinoids following SCI in adult mice does not synergize with a voluntary, aerobic-based rehabilitative intervention, as illustrated by this study. This class of clinically approved drugs does, however, beneficially impact hallmarks of SCI psychopathology. Whereas mice administered GBP consistently engage in the rehabilitation program, control mice lack motivation to participate, especially at chronic time points. Chronic administration of GBP does not attenuate neuroinflammatory gene expression after SCI. Rather, it stimulates hippocampal neurogenesis and TrkB expression in the subgranular zone of the dentate gyrus, likely contributing to suppression of anxiety-like behavior. An external, social motivator is able to effectively rescue participation in control mice and promote forelimb recovery in the chronic phase of SCI. These results underscore the beneficial actions of gabapentinoids on SCI psychopathology and highlight the importance of breaking mental barriers for SCI repair.

SCI not only leads to long-term physical disability, but also to the rapid deterioration of mental wellbeing as the result of increased social isolation, anxiety and depression (Migliorini et al., 2008; Post and van Leeuwen, 2012). Despite progress in the field (Hutson and Di Giovanni, 2019), readily translatable strategies for SCI repair remain elusive. We previously discovered that administration of gabapentinoids promotes structural plasticity and regeneration of ascending and descending pathways after SCI and ischemic stroke in adult mice (Tedeschi et al., 2016, 2022; Sun et al., 2020). Gabapentinoids bind with high affinity and selectivity to Alpha2delta1/2 subunits of voltage-gated calcium channels (Gee et al., 1996; Gong et al., 2001). These drugs are heavily prescribed to treat neurological disorders including neuropathic pain after SCI (Rosner et al., 1996; Fornasari, 2017). Adult mice administered GBP recover forelimb function weeks and months after cervical SCI (Sun et al., 2020). Similarly, early (vs. late) administration of this class of drugs improves motor recovery in SCI individuals (Warner et al., 2017). Given that a multidisciplinary approach is essential for maximization of neurological recovery following SCI (Griffin and Bradke, 2020), we questioned whether gabapentinoids administration may synergize with rehabilitative efforts.

Among others, cardiovascular-based protocols have been proven to enhance neuronal plasticity, promote motor recovery and counteract cardiovascular complications associated with SCI (Krassioukov et al., 2019; Jo and Perez, 2020; Faw et al., 2021). Treadmill training promotes locomotor recovery in experimental models of SCI (Lovely et al., 1986) and in SCI individuals (Wernig and Muller, 1992; Dietz et al., 1994; Thomas and Gorassini, 2005). Studies in rodents, cats and humans indicate that training-dependent upregulation of brain-derived neurotrophic factor (BDNF) promotes neuroplasticity and functional connectivity (de Leon et al., 1998; Gomez-Pinilla et al., 2002; Thomas and Gorassini, 2005). When rehabilitation is begun soon after injury, however, the beneficial response to exercise is disrupted and neurological recovery is delayed (Griesbach et al., 2004). Similarly, the combination of intensive training with regenerative strategies early after injury to the mammalian central nervous system results in aberrant fiber patterns and no functional recovery (Wahl et al., 2014). Following successful completion of preoperative training and after mice had regained weight support and recovered forelimb ground locomotion following injury, we reintroduced SCI mice to voluntary treadmill training at 15 DPI. Our data indicate that the combination of an aerobic-based rehabilitation regimen with GBP administration does not maximize forelimb recovery in mice following cervical SCI.

In light of this finding, direct training of hand and arm function following cervical SCI may prove more effective than whole-body locomotor/cardiovascular training in regaining skilled forelimb function. Along this line, intensive training of hand function in a rat model of experimental SCI has been shown to restore cortical maps and promote recovery of forelimb function in a task-dependent manner (Girgis et al., 2007). Advanced neurotechnologies and robotic systems have recently been developed with the goal of increasing the efficacy of rehabilitation especially after stroke (Staubli et al., 2009; Rodgers et al., 2019; Micera et al., 2020). Similar technological solutions may also be deployed to promote recovery of hand function after cervical SCI in humans. Other potential explanations for the lack of enhanced forelimb recovery observed in mice administered GBP after treadmill training may be found in changes to the signaling cascade underlying the beneficial action of treadmill training. Chronic administration of GBP over a period of 2 weeks does not cause changes in BDNF expression in the ipsilateral dorsal horn of the lumbar spinal cord in animal models of neuropathic pain (Vanelderen et al., 2013). Interestingly, we discovered that chronic administration of GBP enhances hippocampal neurogenesis and TrkB expression in the subgranular zone of the dentate gyrus of the hippocampus at 14 DPI. Given that hippocampal neurogenesis can be stimulated by chronic administration of antidepressants and by voluntary exercise (Malberg et al., 2000; van Praag et al., 2005; Perera et al., 2007) it is likely that GBP administration and treadmill training activate converging signaling pathways, providing a potential explanation as to why their combination does not boost forelimb recovery.

Given that gabapentinoids dampen excitatory synaptic transmission (Tedeschi et al., 2016), it is also possible that the beneficial effect of treadmill training on strengthening synaptic connections along the sensorimotor axis may be lessened in the presence of GBP. Whether gradual interruption of GBP treatment in parallel with increasing loads of rehabilitation training may be necessary to create more favorable conditions to strengthen synaptic connectivity and therefore maximize forelimb recovery after cervical SCI remains to be explored.

To our surprise, voluntary treadmill training is not effective in SCI mice administered vehicle either. When assessing participation toward the rehabilitation protocol, we discovered that SCI mice administered vehicle were not engaged, especially at chronic time points. However, mice administered GBP remain engaged in the training protocol throughout the duration of the study. In addition to sensory and motor disabilities, SCI also prompts a decline in mental health given the increase in social isolation, anxiety and depression that results after injury (Migliorini et al., 2008; Post and van Leeuwen, 2012). A nationwide population-based cohort study has also found that SCI individuals, in particular those <50 years old, have a high risk of anxiety or depression post-discharge (Lim et al., 2017). On the cellular level, elevated levels of pro-inflammatory cytokines associated with depression- and anxiety-like behavior are present following spinal contusion injury in rats (Maldonado-Bouchard et al., 2016; Brakel et al., 2021). Experimentally, depression-like behavior in contused rats can be reversed with antidepressants (Luedtke et al., 2014). GBP may also be administered to SCI individuals conflicted with some forms of mood disorders (Sokolski et al., 1999; Ahmed et al., 2019). Increased BDNF expression and BDNF signaling has been implicated in the mechanisms of antidepressant drugs (Nibuya et al., 1995; Groves, 2007; Casarotto et al., 2021; Castren and Monteggia, 2021). Thus, rehabilitative training after SCI must be equipped to deal with the degradation of mental health that undoubtedly accompanies SCI, especially at chronic time points. Our results indicate that chronic administration of gabapentinoids after SCI may counteract hallmarks of SCI psychopathology. Further dissection of the mechanisms underlying gabapentinoids' action on SCI psychopathology will be an important direction for future investigations.

Mice and humans require social interaction (Simpson and Balsam, 2016); however, the majority of modern rehabilitation programs are designed on the basis of the individual (Zanca et al., 2013). Although further research into optimal logistics and patterns of group therapy is needed (Zanca et al., 2013; Patterson et al., 2016), a considerable number of patients living with CNS injury report that they value group intervention strategies as an opportunity to bond over shared common experiences, reduce social isolation and receive help and feedback to address the new challenges they now face as a result of their injury (Patterson et al., 2016). Thus, we predicted that the reintroduction of an external, social motivator may be able to promote a certain degree of recovery in control mice. At 54 DPI, we introduced a group run strategy into our rehabilitation protocol. Instead of having animals run in individual lanes, we had them run together with their cagemates, while still assessing them on an individual basis. Our results support our hypothesis that an external, social motivator is able to effectively rescue engagement toward the training protocol. Perhaps even more exciting, following implementation of group runs, control mice exhibited increased forelimb recovery, even at chronic time points where recovery is not expected. It is important to note that, although practical, group training is not as effective as daily administration of GBP. Our experimental model of cervical SCI is rather severe. Nonetheless, there is substantial tissue sparing on the opposite side of the spinal cord. In turn, it will be important to determine the contribution of social training in more severe experimental models of SCI, such as a complete injury of the thoracic spinal cord. Altogether, our results highlight the beneficial action of gabapentinoids and group intervention strategies to address SCI psychopathology and promote neurological recovery.

## Data Availability Statement

The raw data supporting the conclusions of this article will be made available by the authors, without undue reservation.

## Ethics Statement

All animal experiments were performed in accordance with the approval of the Institutional Animal Care and Use Committee at The Ohio State University.

## Author Contributions

AT conceived the project and designed the research study. HR, AB, ML, RB, LW, PF, JG, and AT performed the research and analyzed the data. HR and AT wrote the paper. All authors provided feedback and contributed to editing the manuscript. All authors contributed to the article and approved the submitted version.

## Funding

This work was supported by the National Institute of Neurological Disorders (Grants R01NS110681 and R21NS109787 to AT and R01NS118037 to JG) with additional support provided by National Institutes of Health Grants P30 NS104177 and P30 CA16058.

## Conflict of Interest

The authors declare that the research was conducted in the absence of any commercial or financial relationships that could be construed as a potential conflict of interest.

## Publisher's Note

All claims expressed in this article are solely those of the authors and do not necessarily represent those of their affiliated organizations, or those of the publisher, the editors and the reviewers. Any product that may be evaluated in this article, or claim that may be made by its manufacturer, is not guaranteed or endorsed by the publisher.

## Supplementary Material

Supplementary Video 1 Voluntary treadmill training without and with group runs.
